# The risk factors of the progression of rhegmatogenous retinal detachment on patients with the fourteen-day quarantine in the early period of COVID-19 outbreak

**DOI:** 10.1186/s12886-021-01985-5

**Published:** 2021-05-15

**Authors:** Meng Zhao, Jipeng Li, Haicheng She, Ningpu Liu

**Affiliations:** Beijing Tongren Eye Center, Beijing Key Laboratory of Ophthalmology and Visual Science, Beijing Tongren Hospital, Capital Medical University, No1. Dongjiaominxiang Street, Dongcheng District, Beijing, 100730 China

**Keywords:** Rhegmatogenous retinal detachment, COVID-19, Postponed surgery

## Abstract

**Backgrounds:**

The COVID-19 Pandemic has a great impact on hospitals and patients. The 14-day quarantine caused surgery of rhegmatogenous retinal detachment (RRD) postponed. We aimed to explore the risk factors of RRD progression in a group of patients whose surgery was postponed during the top-level emergency response of COVID-19.

**Methods:**

A retrospective case series. Medical records of all consecutive patients with a diagnosis of RRD who underwent a surgical treatment at Beijing Tongren Hospital’s retina service from February 16, 2020, to April 30, 2020 have been reviewed retrospectively. Medical history, symptoms, and clinical signs of progression of RRD were recorded. RRD progression was defined as the presence of either choroidal detachment or proliferative vitreoretinopathy (PVR) progression during the quarantine period. Risk factors were analyzed using the Cox proportional hazards model, survival analysis, and logistic regression.

**Results:**

Seventy-nine eyes of 79 patients met the inclusion criteria and were included in the study. The median time from the patients’ presentation at the clinic to admission for surgery was 14 days (3–61 days). There were 70 cases (88.6%) who did not present to the hospital within 1 week of the onset of visual symptoms. There were 69 (87.3%) macular-off cases at the presentation and 27 (34.2%) cases combined with choroidal detachment. There were 49 (62.0%) cases with PVR B, 22 (27.8%) cases with PVR C, 4 (5.1%) cases with PVR D, and 4 (5.1%) cases with anterior PVR. After the 14-day quarantine, 21 (26.6%) cases showed RRD progression, and 9 cases showed RRD regression at the time of surgery. Neither the time of onset of the visual symptom (*p* = 0.46) nor the time between presentation and admission (*p* = 0.31) was significantly different between the patients with RRD progression and patients without RRD progression. The combination of choroidal detachment (3.07, 1.68-5.60, *p*<0.001) and retinal breaks located posterior to the equator (3.79, 1.21-11.80, *p*=0.02) were factors related to the progression of RRD.

**Conclusions:**

In our study during the COVID-19 outbreak, the RRD progression risk factors included a combination of choroidal detachment and retinal breaks posterior to the equator. Ophthalmologists should schedule the surgeries for RRD patients with these signs as soon as possible.

## Background

The outbreak of the highly contagious coronavirus disease (COVID-19) has a significant impact on health services worldwide. The early impact of eye care has been reported in different countries [1]. Most elective surgeries were postponed in response to the Pandemic except for urgent surgeries [2–4]. In China, Beijing upgraded its emergency response to top-level from February 16, 2020, 3 days after ten COVID-19 cases were confirmed, and ended April 30, 2020. During this period, surgeries for rhegmatogenous retinal detachment (RRD) were carried out only in patients who had a negative screening result for COVID-19. Since there was no laboratory test of the COVID-19 virus in the early days of the Pandemic, the COVID-19 screening in our center included a 14-day self-quarantine with daily self-reported normal body temperature, as well as a normal blood cell count and pulmonary CT scan before admission. During the period, the RRD patients could only visit the emergency room for diagnosis and wait at least14 days before surgery.

Several authors have discussed the ethical impact of delivering treatments to patients in need since the beginning of the COVID-19 outbreak [1, 5]. The EUROCOVAT group has reported changes in ophthalmology training [6], elective cataract surgeries [7], and corneal donors [8]. The treatment priority should be given to patients with a high risk of morbidity due to treatment delay over the patients’ slowly progressed condition. A framework has been reported in patients receiving intravitreal injections suffering from sight-threatening conditions when the available resources are limited, precluding all patients’ accommodation [5]. RRD is a sight-threatening ocular disease requiring surgical intervention [3]. It can lead to irreversible visual damage [9] and should be treated in time [10, 11]. However, the 14-day quarantine made it is impossible to treat the RRD in time. To date, the changes of RRD characteristics over the 14-day quarantine have not been reported. Previous reports focusing on RRD progression found that prolonged pre-surgery waiting time [12], pseudophakia [13], and bullous configuration of detachment [14] are related to RRD progression. There are no studies regarding the onset of new possible risk factors on affected patients undergoing 14-day quarantine during the COVID-19 outbreak.

The aim of the present study was to investigate the progression of RRD and its related risk factors after a 14-day quarantine during the early period of COVID-19 outbreak.

## Methods

We retrospectively reviewed all the consecutive cases of RRD who underwent surgery from February 16, 2020, to April 30, 2020, in Beijing Tongren Eye center. Exclusion criteria included records of patients who were not operated due to ocular or systemic contradictions. This study was approved by the Ethics Committee of Beijing Tongren Hospital and adhered to the tenets of the Declaration of Helsinki.

All patients were registered for surgery at the time when they were diagnosed as RRD. Patients underwent visual acuity (VA) examination, intraocular pressure test, slit-lamp examination, and indirect fundus ophthalmoscopy with scleral indentation at outpatient clinics and were asked to report their daily temperature and quarantine in Beijing. Cases with choroidal detachment were treated with either local or systemic steroid [15]. After the 14-day quarantine, patients were screened for the COVID-19, taking laboratory tests and receiving ocular and systemic examinations for surgery. We collected the preoperative characteristics of the patients, including age, gender, pre-surgery waiting times (from visual symptom to surgery), quarantine time (from being diagnosed to surgery), history of previous eye trauma, previous surgical history, preoperative VA, lens status, the extent of retinal detachment, location of retina breaks, proliferative vitreoretinopathy (PVR), presence of choroidal detachments and/or pathological myopia (PM), and congenital vitreoretinal abnormalities such as coloboma and familial exudative vitreoretinopathy. The changes of RRD, in particular the development and progression of choroidal detachments and PVR, were recorded after quarantine as compared to the record of the first visit. PVR progression was defined as the growth of membranes on both surfaces of the detached retina and on the posterior surface of the detached vitreous gel [16] and its severity was classified as either posterior or anterior PVR [17]. Choroidal detachment was defined as the presence of low intraocular pressure, anterior chamber inflammation, choroidal detachment found by either indirect ophthalmoscopy or B scan [18]. PM was defined as the presence of myopic fundus lesions in addition to a high degree of myopia [19]. RRD progression was recorded as at least one of the following conditions developed after quarantine as compared to the initial presentation: 1) development of choroidal detachment; 2) progression of PVR; 3) the extent of retinal detachment progressed from 1–2quadrants to 3–4 quadrants.

### Statistical analysis

Statistical analysis was performed using R version 3.20 (http://www.R-project.org). Patient characteristics were retrieved from their medical charts and recorded in Epidata Entry Clientversion2.0.3.15 (http://epidata.dk). Mean and standard deviation (SD) were calculated for continuous variables with a normal distribution. Median with interquartile range (IQR) was calculated for continuous variables with a non-normal distribution. T-test or Mann-Whitney U test was carried out for continuous variables. Chi-square test or Fisher’s exact test was carried out for discrete data. The standard level of significance used to justify a claim of a statistically significant effect is 0.05. The Cox proportional-hazards model was used to investigate the association between patients’ quarantine time and several characteristics that may be related to RRD progression. The survival analysis, Kaplan-Meier curve, and log-rank test were performed on the related factors. The binary backward stepwise logistic regression model was carried out to explore the potential risk factors. One variable was included or excluded from the model each time by comparing the Akaike information criterion (AIC) value, and the model with the lowest AIC was chosen.

## Results

Seventy-nine eyes of 79 patients were enrolled in the study, with a majority of male patients (70.9%). Two patients were excluded due to systemic contradiction to surgery. The average age was 49.8 ± 15.7 (12–74) years old. The median quarantine time was 14 days (3–61, IQR 12). The median time between the onset of symptoms and operation was 28 days (7–336, IQR 35). There were 70 (88.6%) patients who did not present to the hospital within 1 week after the onset of their visual symptoms.

### Basic characteristics at presentation

There were 45 (57.0%) patients with primary RRD, 27 (34.2%) patients combined with choroidal detachments, 5 (6.3%) patients having a failed RRD surgery (1 had scleral buckle and 4 had pars plana vitrectomy), and 2 (2.5%) patients having coloboma with normal axial length.

There were 18 (22.8%) pseudophakic eyes and 29 (36.7%) eyes with PM. Ten (12.7%) eyes had a past surgical history of retinal detachments, including 4 (5.1%) eyes with scleral buckle and 6 (7.6%) eyes with vitrectomy. Having had Six patients had a previous history of PPV (four patients had recurrent RRD, one patient had vitreous hemorrhage due to branch retinal vein occlusion, one patient had a macular hole) while five patients had a history of scleral buckling (one patient had recurrent RD who developed re-detached retina 4 months after the initial SB procedure, four patients failed to retina attachment after the first SB procedure). There were 44 (55.7%) patients whose preoperative VA was less than 0.02, 28 (35.5%) patients whose preoperative VA was between 0.02 and 0.4, and seven (8.9%) patients whose preoperative VA was equal to or greater than 0.5.

There were 42(53.2%)patients with four quadrants RD. There were 68(86.1%)patients with a macular-off RD.

There were 49 (62.0%) patients with PVR B, 22 (27.8%) patients with PVR C, four (5.1%) patients with PVR D, and four (5.1%) patients with anterior PVR. The prevalence of PVR C-D and anterior PVR was higher in patients with RRD-CD than patients with RRD (44.1, 31.1%, *p* = 0.01).

Thirty-seven (46.8%) patients’ retinal breaks were located anterior to the equator, 38 (48.1%) patients’ retinal breaks were located posterior to the equator, and four (5.1%) patients had a macular hole (one of them combined with superior tear which was posterior to the equator). Forty-six patients (58.2%) had retinal break located above 10–2 o’clock, 16 patients (20.2%) had a retinal break at 3 or 9 o’clock, 13 patients (16.5%) had retinal break located at 4–8 o’clock, three patients had a macular hole (3.8%), one patient (1.3%) had a macular hole and superior tear. RRD-CD prevalence was higher in patients with retinal breaks located posterior to the equator than patients with retinal breaks located anterior to the equator (55.9, 22.2%, *p* = 0.01).

### Changes of RRD at admission

Twenty-one (26.6%) eyes had a progression of RD after quarantine. Among them, 16 RRD-CD eyes had a progression of PVR (four eyes from C1 to D2, six eyes from C1 to C3, and two recurrent eyes with anterior PVR), and five RRD eyes developed CD (one had a simultaneous progression of PVR). Nine (11.4%) eyes had RD regression, while 49 (70.6%) eyes had no significant RD progression, including 11 RRD-CD eyes.

Fifty-seven patient (72.2%) received PPV, while 22 patients (27.8%) received scleral buckling. Among the patients who received PPV, ten patients had preoperative posterior vitreous detachment, 25 patients whose PVD was easily induced during vitrectomy, and 28 patients had sticky vitreous, which was hard to peal during vitrectomy.

### Factors that may be related to the progression of RD

We divided the patients into two groups based on whether RD progressed at admission (Table 1). There was a significant difference between the two groups in terms of the following factors: gender (*p* = 0.04), the combination of CD (*p* < 0.001), previous history of vitrectomy (*p* = 0.04), location of retinal breaks (*p* < 0.001), macular hole (*p* = 0.03), macular detachment (*p* = 0.05), sticky vitreous during vitrectomy (*p* < 0.001), and VA distribution (*p* = 0.04). There was not a significant difference in quarantine time (*p* = 0.46) or the time between the onset of symptoms and presentation (*p* = 0.31) in the two groups (Table 1).

**Table 1:** Initial characteristics of RRD patients enrolled

	Patients with RD progression (21)	Patients without RD progression(58)	*p* value
Age (mean ± SD)	51.1 ± 12.2	49.3 ± 16.9	0.62
Gender (male, n, %)	19, 90.5%	37, 63.8%	0.04
Time between diagnosis and surgery (median, range, day)	18 (3–61)	13 (11–75)	0.46
Time between onset of symptom and surgery (median, range, day)	28 (7–84)	28 (7–336)	0.31
Diagnosis			< 0.001*
RRD (n,%))	2, 9.5%	42, 72.4%	
RRD-CD(n, %)	14, 66.7%	14, 24.1%	
Recurrent RRD(n, %)	3, 14.2%	2, 1.7%	
Combined coloboma(n, %)	2, 9.5%	0	
RRD-CD on steroid treatment (n, %)	10, 71.4%	12, 85.7%	0.67
pseudophakic(n, %)	7, 33.3%	11, 19.0%	0.22*
PM (n, %)	8, 38.1%	21, 36.2%	1*
Previous PPV(n, %)	4, 19.0%	2, 3.4%	0.04 *
Previous SB(n, %)	1, 4.8%	4, 6.9%	1 *
Location of retinal break			0.03
Anterior to equator (n, %)	1, 4.8%	36, 62.1%	< 0.001
Posterior to equator (n, %)	17, 81.0%	21, 36.2%	
Macular hole(n, %)	3, 14.3%	1, 1.7%	0.05
Position of retinal break			
10–2 o’clock(n, %)	13, 59.1%	33, 57.9%	1.0
3 or 9 o’clock(n, %)	5, 22.7%	11, 19.3%	0.76
4–8 o’clock(n, %)	5, 22.7%	8, 14.0%	0.50
Macular hole with superior tear(n, %)	1, 4.8%	0, 0%	0.27
Macular hole(n, %)	1, 4.8%	2, 3.5%	1.0
PVR			0.35
B(n, %)	12, 57.1%	37, 63.8%	
C(n, %)	5, 23.8%	17, 29.3%	
D(n, %)	2, 9.5%	2, 3.4%	
anterior PVR(n, %)	2, 9.5%	2, 3.4%	
Macular-off(n, %)	21, 100%	48, 82.8%	0.05
PVD (n, %)			< 0.001*
Presurgery VA			0.04
Less than 0.02(n, %)	14, 66.7%	21, 36.2%	
[0.02–0.1) (n, %)	7, 33.3%	30, 51.7	
> =0.5(n, %)	0	7, 12.1%	

*RRD* rhegmatogenous retinal detachment, *RRD-CD* rhegmatogenous retinal detachment with choroidal detachment, *PPV* pars plana vitrectomy, *SB* scleral buckling, *PVR* proliferative vitreoretinopathy, *PVD* posterior vitreous detachment; VA: visual acuity

**Fig. 1:**
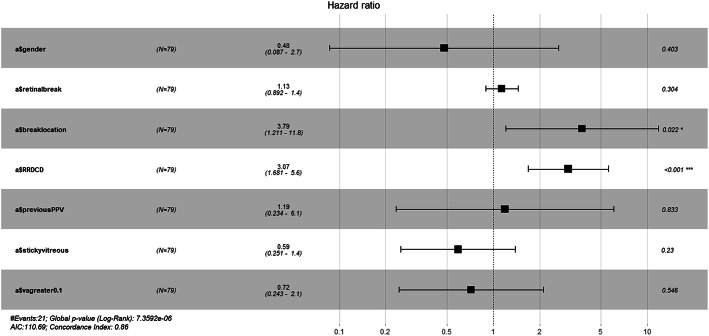
The tree-plot of Cox proportional-hazards model for RD progression. The patients with RRD-CD at presentation were 3.07 times more likely to have RD progression (1.68–5.61, *p* < 0.001) than patients without CD; patients with retinal breaks located posterior to the equator were 3.79 times more likely to have RD progression (1.21–11.84, *p* = 0.02) compared to patients with retinal breaks located anterior to the equator (Wald test F = 27.0, *p* < 0.001, LogRank test F = 45.1, *p* < 0.001)

**Fig. 2:**
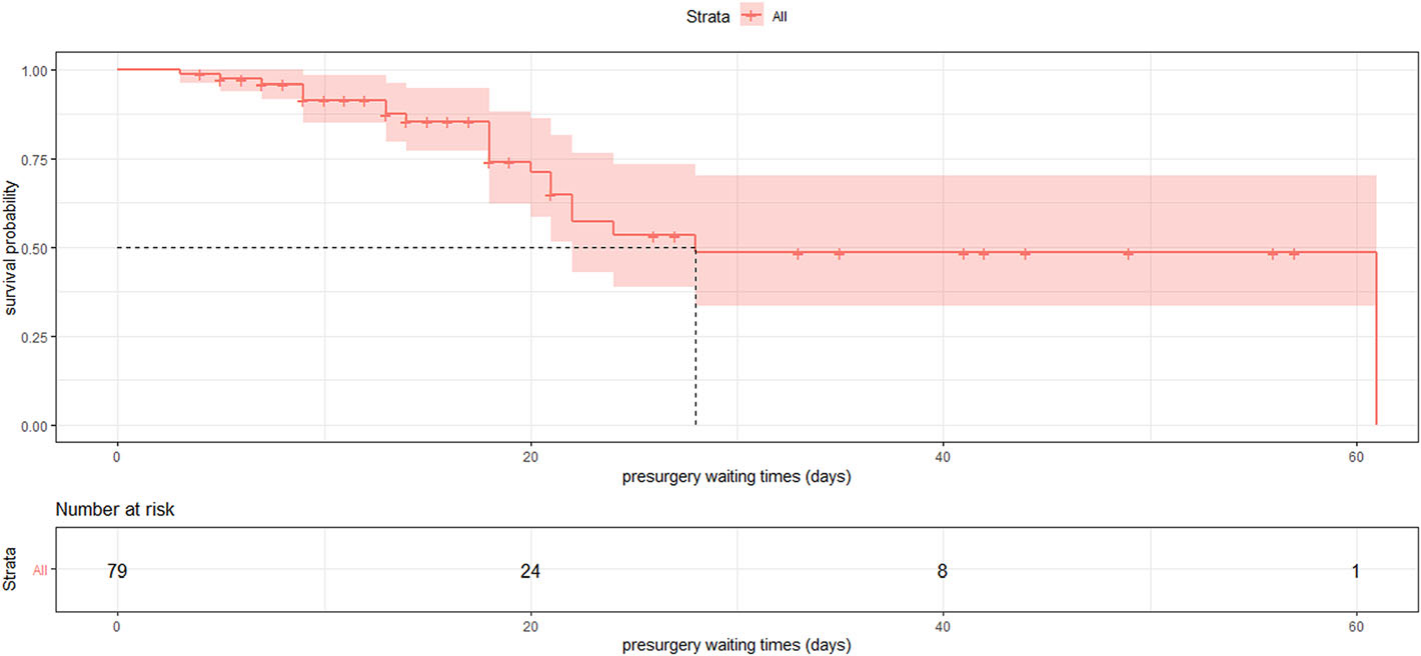
Kaplan-Meier survival analysis curve for the RD progression. The median survival time for RD progression was 28 days after the onset of symptoms

The Cox proportional-hazards model showed that patients with RRD-CD at presentation were 3.07 times more likely to have RD progression (1.68–5.61, *p* < 0.001) than patients without CD; patients with retinal breaks located posterior to the equator were 3.78 times more likely to have RD progression (1.21–11.84, *p* = 0.02) compared to patients with retinal breaks located anterior to the equator (Wald test F = 27.0, *p* < 0.001, LogRank test F = 45.1, *p* < 0.001). The median time for RD progression was 28 days after the onset of symptoms (Figs. 1 and 2). The log-rank test in survival analysis showed that the median time for RD progression was 13.5 days in patients with macular hole, 22 days in patients with retinal breaks located posterior to the equator, 14 days in patients with giant tears, 18.5 days in patients with unattached retina after surgery, 22 days in RRD-CD patients, and 18 days in patients with sticky vitreous.

The logistic regression analysis showed that female (25, 2.04–1000, *p* = 0.03), combination with CD (5.22, 2.18–17.66, *p* = 0.001), and retinal breaks located posterior to the equator (17.91, 3.44–224.56, *p* = 0.004) were factors that may be related to RD progression (AIC = 52.23, AUC = 0.918).

## Discussion

The 14-day quarantine during the early period of the COVID-19 Pandemic provided us the chance to observe the short natural course of RRD when the surgery had to be delayed. We found that 26.6% of patients had RD progression, while 11.4% of patients had RD regression, 70.6% of patients had no significant RD progression after the quarantine. We further investigated the initial characteristics that may be related to the RD progression.

Previous reports focusing on RD progression find that prolonged presurgery waiting time is related to the development of macular-off RD [12], irreversible macular damage [20–22], PVR progression [23], and development of CD [24]. We chose PVR progression, RD’s progression, and CD’s development as the signs for RD progression. Since the prevalence of macular detachment (86.1%) in our group of patients was much higher than what was reported in previous studies [10, 11], which focused on RD’s progression from macular-on to macular-off, we could not use the development of macular-off as a sign for RD progression.

CD is related to retinal detachment surgery failure [25], and its prevalence is 8.6–18.79% [26, 27] in Chinese RRD patients. We had a much higher prevalence of RRD-CD in our group of patients at 34.2%. The prevalence of PVR C-D is higher in RRD-CD patients at 28–66.9% [28, 29] compared to RRD patients in previous reports. PVR progression was found in 16 RRD-CD patients. We found a similar result of PVR C-D at 44.1% in RRD-CD patients and 33.1% in RRD patients. We found a retinal break located posterior to the equator was related to the RRD-CD development, similar to previously reported [26]. Corticosteroids are widely used preoperatively in addition to surgery to treat patients affected by RRD-CD and play an essential role in preparing patients affected by RRDCD for surgery by reducing the permeability of choroidal blood vessels, inhibiting inflammatory reactions and cellular proliferation [15]. Our result showed RRD-CD patients were much more likely to experience RD progression shortly after diagnosis, even when they were under steroid therapy. The median time for RD progression in RRD-CD patients was 22 days after the onset of the symptom. Our result suggested that patients with RRD-CD should be operated on without delay in RD’s fast progression cases. The effect of steroid therapy on the progression of RRD-CD should be further explored in the RRD-CD patients’ cohort with a large sample size involving more RRD-CD patients with or without steroid treatment.

PVR is the most common cause of RRD surgery failure [16, 25, 30, 31]. PVR progression is related to the following conditions: a giant tear, a long course of RRD, vitreous hemorrhage, pseudophakic eye, the combination of CD, gas tamponade, and cryotherapy [32, 33]. The chance of developing PVR B-C has been reported to be higher in patients whose presurgery waiting times are longer than 40 days [23]. The prevalence of PVR C-D and anterior PVR in our study was 38.0%. It was similar to what was previously reported at 12.9–21.6% [34] in patients with scleral buckling and 26.9–41.6% [23, 34, 35] in patients with PPV. The initial presentation of PVR in our group of patients was not related to RD progression. The reasons may be related to the fact that the presurgery waiting time was too short to observe PVR progression. In other words, except for patients with CD, the presence of PVR was not related to short-time RD progression.

In addition to the presence of CD and the location of a retinal break, the survival analysis found that the median survival time for RD progression was short in the following conditions in survival analysis: macular hole (13.5 days), giant tear (14 days), combining with coloboma (6 days), and recurrent RD (18.5 days). We failed to show the relationship between the factors mentioned above to RD progression due to the small sample size. We still need to pay extra attention to RRD patients with the conditions mentioned above in the case of RD progression.

The limitation of the study was due to the retrospective character and the limited case number. We failed to report the patients’ refractive status and corrected VA due to the lack of refraction examination during the Covid-19 Pandemic. The prolonged presurgery waiting time may lead to a high CD, PVR C-D, and macular-off prevalence. We failed to show why the initial high prevalence of 4 quadrants RD and macular-off patients at outpatients clinics. The reluctance to visit outpatients clinic and the limited outpatients’ service during the Covid-19 Pandemic may take account for it. Since there was a high prevalence of 4 quadrants RD in our group of patients, the presence of more challenging cases at the initial presentation makes it impossible to progress to a more severe condition in a short follow-up time. More cases with newly developed RRD with short presurgery waiting time should be involved to investigate the risk factors for RD progression. Also, we did not report the outcomes of surgeries. The pars plana vitrectomy [36] and scleral buckling [37] are two major effective procedures for treating RRD with a high anatomical success rate. We did not include the impact of the prolonged presurgery waiting time on the procedure choice. We can not show the impact of prolonged presurgery waiting time on the prognosis of RRD either. The intravitreal injection of steroid service was postponed during the Covid-19 Pandemic, and we could not show the effect of intravitreal steroid on the progression of RRD.

## Conclusion

We have reported a group of RRD patients with a high prevalence of PVR C-D, CD, and macular-off who underwent surgery during the COVID-19 Pandemic. After the quarantine, some of the patients had RD progression. Ophthalmologists should pay more attention to RRD patients with CD or retinal breaks located posterior to the equator in case of RD progression shortly after the diagnosis.

## Data Availability

The datasets used and/or analyzed during the current study are available from the corresponding author on reasonable request.

## Abbreviations

AIC: Akaike information criterion
CD: choroidal detachment
COVID-19: coronavirus disease
BCVA: visual acuity
IOP: intraocular pressure
IOL: intraocular lens
MH: macular hole
PM: pathological myopia
PVD: posterior vitreous detachment
PVR: proliferative vitreoretinopathy
ROC curve: receiver operating characteristic curve
RRD: rhegmatogenous retinal detachment
RD: retinal detachment
SD: standard deviation
